# Bouncing Back: The Psychosocial Benefits of a Community-Based Exercise Program for Children with Non-Severe Burns

**DOI:** 10.3390/ebj6010009

**Published:** 2025-02-17

**Authors:** Dinithi Atapattu, Victoria M. Shoesmith, Eva Kierath, Mark W. Fear, Fiona M. Wood, Lisa J. Martin

**Affiliations:** 1 Fiona Wood Foundation, Burns Unit, Fiona Stanley Hospital, MNH (B) Main Hospital, Level 4, 102-118 Murdoch Drive, Murdoch, WA 6150, Australiafiona.wood@health.wa.gov.au (F.M.W.); 2 Burn Injury Research Unit, University of Western Australia, 35 Stirling Highway, Crawley, WA 6009, Australia; 3 Burn Service of Western Australia, Fiona Stanley Hospital, MNH (B) Main Hospital, Level 4, Burns Unit, 102-118 Murdoch Drive, Murdoch, WA 6150, Australia

**Keywords:** burn injury, pediatric, rehabilitation, exercise, community, psychosocial, physical, psychological, intervention, trampolining

## Abstract

Burns significantly impact children’s physical and psychosocial recovery, even in cases of non-severe injuries, leading to long-term health and mental health risks. This study explores the psychosocial benefits of a community-based exercise program for children recovering from burn injuries, addressing concerns such as anxiety, reduced physical activity, and social challenges. A pre-test–post-test design assessed the effects of an 8-week community-based trampoline exercise intervention on psychosocial outcomes in children and their caregivers. No significant or clinically meaningful physical improvements were observed across measures such as MET score, grip strength, BMI percentile, or heart-rate recovery despite a significant improvement in trampolining performance (*p* < 0.0001). Psychosocial outcomes showed improved child emotional function (PedsQL, *p* = 0.024) as reported by parents, though children’s self-reported emotional function and Child PTSD Symptom Scale (CPSS) scores remained unchanged. Parent-reported strengths and difficulty scores for the child remained stable over time but were higher than population norms for hyperactivity and emotional difficulty. Parental post-traumatic stress symptoms decreased significantly over time (*p* = 0.050), with reductions in avoidance (*p* = 0.009), hypervigilance (*p* = 0.007), and intrusion scores (*p* = 0.026). Children significantly improved their trampolining performance, while parents reported enhanced emotional function for their child. However, children’s self-reports did not reflect these emotional improvements.

## 1. Introduction

Burns are a major global public health concern, causing an estimated 180,000 deaths every year, and are a leading cause of morbidity [1]. As burn treatments improve, mortality rates decline, and attention shifts to optimizing recovery through diverse comprehensive aftercare packages that focus equally on the physical and psychosocial needs of the survivors [2,3]. In Australia and New Zealand in 2022–2023, there were more than 2400 patients admitted to hospital for acute burn care, and approximately 25% of these were children and adolescents [4].

Following severe burns, return to exercise can be limited due to loss of physical function caused by scarring [5], and muscle wasting caused by hypermetabolic changes that can last many months or years [6,7]. However, there are also impacts for those with non-severe burns [8]. In a qualitative study, children with non-severe burns expressed concerns about disruptions to their routine participation in sports and expressed anxiety about confidently re-engaging in sports [9]. This can be compounded by vicarious trauma experienced by their parents [10], which can lead to hypervigilance around perceived dangers to their child [11,12].

Non-severe pediatric burn injury (<20% TBSA) has long-term impacts [13] with an increased risk of readmission for physical health conditions, including musculoskeletal disorders [14], and strength trajectories negatively impacted, especially in young girls [15]. In addition, readmissions for mental health conditions are between two and five times greater for children who have sustained a burn injury compared to an uninjured cohort [16], and 16% of children who have experienced a non-severe burn report psychosocial quality-of-life (QoL) scores that are below the minimum accepted threshold at 3 months post burn [17]. Supervised exercise programs have been shown to benefit rehabilitation in both adult and pediatric severe-burn survivors [18].

Physical activity is an important part of pediatric rehabilitation, including children recovering from burn injuries. In the pediatric burn population, children can experience long-term deficits in muscle strength and aerobic capacity, as well as impairments in day-to-day activities. Studies have shown that pediatric burn survivors suffer from reduced muscle mass, loss of strength, and limitations in pulmonary and aerobic capacity compared to their healthy counterparts [19,20]. Loss of strength appears to occur regardless of burn severity. Grip strength, for example, was found to be impaired following non-severe pediatric burn, whether or not the hand had been involved in the burn injury. Female children, especially those burned at a younger age, were found to experience higher levels of strength impairment at several years post burn, suggesting that the trajectory for strength acquisition across childhood is lowered from the time of injury [15]. These deficits underscore the need for tailored rehabilitation programs that address both strength and fitness. Other research has found that supervised exercise programs can improve muscle strength, lean body mass, and overall quality of life in pediatric burn survivors [7,21]. Aerobic exercises, resistance programs, and even music-based rehabilitation have shown positive outcomes in restoring muscle function and range of motion [22].

Physical activity programs have been found to improve QoL, psychosocial health and mental health, as well as improve physical problems faced by children surviving a burn [23,24]. Post-traumatic stress responses and scarring can affect social interactions [25,26] and children may worry about the appearance of their scars and how other people may react [9]. Engaging in community programs where they can connect and relate with other children with similar health concerns could be beneficial in boosting self-esteem and motivation [27]. To our knowledge, this innovative study is the first to evaluate community-based exercise programs for psychosocial recovery in children with non-severe burns. The aim of this study was to explore the potential psychosocial benefits of a community-based exercise program for children who had experienced a burn injury.

## 2. Materials and Methods

This was an exploratory, longitudinal, pre-test–post-test study assessing the effects of an 8-week exercise intervention on psychosocial outcomes in children, with measurements taken at baseline (pre-test), immediately after the intervention (post-test), and 3 months post completion (follow-up). Ethics approval was gained from the Institutional Ethics Committee (RGS0000004999). Patients were eligible for the study if they were 4 years or older and had received acute burn care treatment at Perth Children’s Hospital. Potential participants were screened for eligibility when attending their routine outpatient appointment. They were invited to the study, fully informed consent was obtained, and those who were interested and able to attend the trampolining gym were recruited. Those who had significant psychiatric morbidity, social issues requiring psychosocial intervention, or physical comorbidities limiting participation in physical activity were excluded.

### 2.1. Demographic and Clinical Data Collection

Demographic information collected included age at the time of injury (years), sex at birth (male, female), and postcode of residence. Clinical data were collected from medical records and included burn type, the bodily location of the burn, the percentage of total body surface area burned (TBSA%), burn depth (superficial partial, mid-dermal, deep, full thickness), and acute wound closure surgical treatment received (split-thickness skin graft (SSG), ReCell^TM^, a combination of these, or other). Social information collected included marital status of parents, languages spoken at home other than English, indigenous status, and the family’s country of origin.

### 2.2. Measures

Psychosocial quality of life and physical measures were collected at baseline (Visit 1), then at two post-intervention time points: immediately after the completion of the trampoline program (Visit 2), and at 3 months post completion (Visit 3). At all visits, the following measures were collected.

#### 2.2.1. Psychosocial Outcomes

The Strengths and Difficulties Questionnaire (SDQ) was used to assess emotional and behavioral functioning. The SDQ is a 25-item screening tool that identifies emotional symptoms, behavioral problems, hyperactivity tendencies and social behaviors and can be reported by parents or teachers for children 4 years and older. The SDQ has been validated in other studies with pediatric burn patients [28] and norms are available for Australian children [29]. Parents reported this for all children.

The Child PTSD Symptom Scale (CPSS) was administered to children 8 years and older to measure post-traumatic stress symptoms. The Child PTSD Symptom Scale (CPSS) assesses PTSD diagnostic criteria and symptom severity among children and adolescents aged 8–18 years. The scale includes 17 symptom and 7 functional impairment items. The symptom items are divided into three subscales: re-experiencing, avoidance, and hyper-arousal behaviors, each measured on a 4-point frequency scale from 0 (not at all or only at one time) to 3 (5 or more times a week/almost always). The functional impairment items are measured as absent or present [30]. Eighteen children were 8 years or older and self-reported this measure.

Parents completed the Impact of Event Scale—Revised (IES-R) to measure their own post-traumatic stress symptom responses. The IES-R has 22 items and is used to measure post-traumatic stress symptoms [31]. Parents self-report this measure, and it takes 5–10 min to complete. All parents reported this measure for themselves.

Optional hair samples were collected from both children to analyze cortisol levels as an objective measure of post-traumatic stress symptoms, with parental consent.

#### 2.2.2. Quality of Life

The Pediatric Quality-of-Life Scale Version 4 (PedsQL^™^) a 23–25 item measure, was used to assess general physical, emotional, social, and school function domains. There are age-specific adaptations of the measure, allowing both parents and children (aged 5 years and older) to complete the assessment. This measure has been validated in various areas of health care, including burns [17,32,33].

#### 2.2.3. Physical Outcomes

Physical activity was measured using the Children’s or Youth’s Physical Activity Questionnaire (CPAQ/YPAQ) for participants aged 5 years and older. This questionnaire evaluates typical daily physical activity and gives scores of Metabolic Equivalence of Tasks (MET score) [34]. A total MET score was calculated based on the activities the child completed during the previous week.

The 10-bounce time was recorded at Visits 1 and 2 to measure improvements in endurance, control, and consistency. It refers to the total time taken to perform 10 consecutive straight bounces on a trampoline. A longer 10-bounce time indicates better height control and consistency, as the child is able to maintain each bounce for a longer period of time without losing height or control. It is used as a metric to assess the skill level and performance of a trampolinist, focusing on their ability to maintain balance, rhythm, and height across multiple bounces.

Grip strength was recorded as an indicator of strength. This measure has been validated in pediatric burn patients [15] and healthy pediatric norms are available [35]. Additionally, a 3 min step test was used to assess aerobic fitness at baseline, post intervention, and at 3 months follow-up. This test has been validated in children as young as 6 years old [36]. Height and weight were recorded, and the BMI percentile was calculated. This measure compares a child’s Body Mass Index (BMI) to that of other children of the same age and sex, using growth charts established by the US Center for Disease Control (CDC). The percentile indicates the relative position of the child’s BMI among peers; for example, a child at the 85th percentile has a BMI greater than 85% of children of the same age and sex. This metric accounts for variations in growth patterns, helping to assess whether a child is underweight (<5%), normal weight (5–<85%), overweight (85–<95%), or obese (95% or higher) based on standardized growth norms.

### 2.3. Intervention

The intervention consisted of a community-based trampolining program, specifically designed by an expert trampoline coach and an exercise physiologist to suit the age and physical ability of participants. Children attended group sessions lasting at least 60 min, with a total of 8 h of instruction spread across multiple sessions. The trampoline sessions aimed to improve physical fitness, psychosocial functioning, and quality of life post burn injury. They consisted of a structured program in which participants could progress at their own pace. Starting with simple straight, pike, and straddle jumps, and increasing in complexity to turns, drops, and somersaults. Extra equipment such as airbags and rigs could be used to assist where appropriate.

### 2.4. Analysis

Descriptive summary statistics were used to describe the demographic, clinical, and outcome data for the participants. For continuous variables, medians and interquartile ranges were reported. For categorical variables, proportions or percentages were used. Non-parametric testing methods were applied to both categorical and continuous data across two and three time points due to the small sample size and the non-normal distribution of the data. For continuous data, the Wilcoxon signed-rank test was used to compare outcomes between two time points, such as pre-test versus post-test. When comparing continuous data across three time points (e.g., pre-test, post-test, and follow-up), the Skillings–Mack test was applied. This robust test used a conservative approach to avoid false-positive results and was able to use all available data (missingness was minimal: grip, height, weight, and step test data were not collected from one participant at Visit 3; all other data were present). For the SDQ, the means and standard deviations were compared to Australian norms [26]. Cortisol was extracted using a methanol extraction procedure from the hair samples and analyzed in duplicate using a commercially available ELISA assay (Stratech, Sydney, Australia). All data analyses were performed using Stata 17.0.

## 3. Results

### 3.1. Patient Characteristics

Of the 24 participants, 66% were female (n = 16), with a median age of 9.5 years (IQR 2.5, range 4–14). Nine patients spoke another language in the home, one patient was Aboriginal. Fourteen families were from Australia or New Zealand, seven from Asia, two from the United Kingdom, one from South America. Fifteen children had primary caregivers who were married or partnered, and nine had single primary caregivers.

The median burn surface area was 1.75% TBSA (IQR 3.25, range 1–10). At their deepest point, six were full-thickness burns, eight were deep, and the remaining ten were superficial or partial thickness. Fourteen patients (58%) required acute surgery for wound closure, with one of those requiring a regraft. Acute surgery was ReCell^TM^ l (n = 6), split-thickness skin graft (n = 1), or split-thickness skin graft and ReCell^TM^ (n = 7). The median time post burn was 1 year (IQR 4, range 0–8). Scald was the most common injury type affecting 58% (n = 14) of participants, and 25% (n = 6) were due to contact burn, with 2 friction burns, 1 electrical, and 1 chemical burn.

### 3.2. Psychosocial Outcomes

Median SDQ scores did not change over time (Table 1).

However, the mean scores differed to Australian norm mean scores for total difficulties, emotional function, and hyperactivity. All of these domains were higher at Visit 1, with total difficulties settling in line with Australian norms at Visit 2 (Table 2).

Parent-reported emotional function: For the PedsQL, there was a significant improvement between Visit 1 and Visit 2 (*p* = 0.011) and across all visits (*p* = 0.024). Parents believed their children had poor emotional functioning at Visit 1, and that it improved over time. This was also reflected in the parent-reported SDQ emotional difficulty scores when compared to the Australian norms, which were significantly higher in this study at Visit 1 (*p* = 0.0048), no different to norms at Visit 2 (*p* = 0.4), but higher than norms at Visit 3 (*p* = 0.018). Child-reported emotional function (PedsQL): children did not agree with their parents, showing no significant changes in their self-reported emotional function across visits (*p*-values all >0.1). CPSS scores remained unchanged.

Parental post-traumatic stress symptoms (IES-R): Significant reductions in parents’ avoidance, hypervigilance, and intrusion scores, indicating reduced stress across the intervention that was sustained over time: avoidance score: significant from Visit 1 to Visit 2 (*p* = 0.024) and across visits (*p* = 0.009). Hypervigilance score: significant from Visit 1 to Visit 2 (*p* = 0.005) and across visits (*p* = 0.007). Intrusion score: significant from Visit 1 to Visit 2 (*p* = 0.020) and across visits (*p* = 0.026) (Table 1).

### 3.3. Physical Outcomes

No significant physical benefits were observed across physical measures (MET score, grip strength, BMI percentile, heart-rate recovery). Grip strength scores were significantly lower than healthy age-appropriate norms at all visits (*p* < 0.0001). Median values remained stable, but a significant increase was detected between Visit 2 and Visit 3 (*p* = 0.025), and grip strength scores at Visit 3 were similar to healthy norms (*p* = 0.3) BMI percentile showed significant overall change across all visits (Skillings–Mack *p* = 0.021), but no significant pairwise changes, suggesting general variability rather than consistent improvement. The PedsQL physical function scores were within healthy norms and were stable (Table 2). Trampolining performance: There was a significant improvement in bounce time from Visit 1 to Visit 2 (*p* < 0.0001), indicating that children became better at trampolining across the intervention.

### 3.4. Trampolining Skills Progress

**Figure 1:**
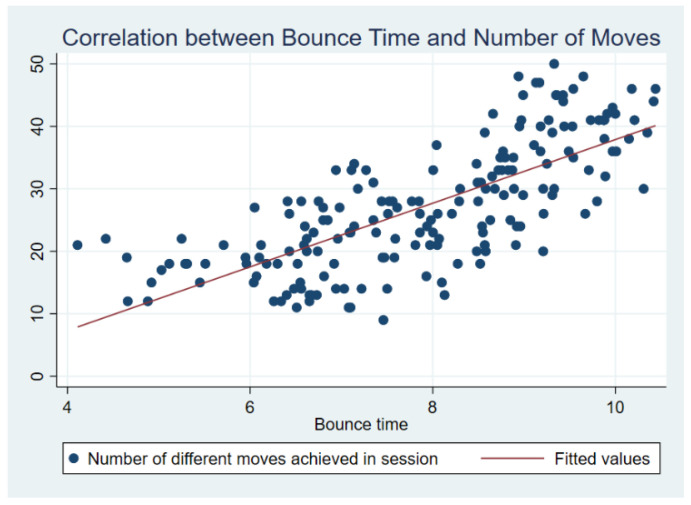
Trampolining skill progress.

There was a strong correlation between the different types of bounce moves achieved and the 10-bounce time (Spearman’s rho 0.7672, *p* < 0.0001) as the number of achieved moves increased; this is reflected in the improvement of the 10-bounce time scores. All participants completed eight sessions. The eight sessions were completed across a median of 9 weeks (range 5–16) (Figure 1).

### 3.5. Cortisol Levels

**Figure 2:**
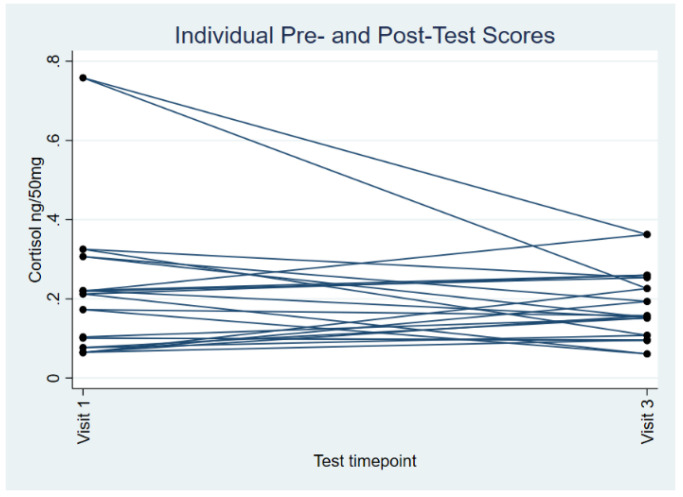
Pre- and post-test hair cortisol.

The optional hair samples were obtained for 12 children at both Visits 1 and 3. The intra-assay variability was 4.1% and inter-assay variability was 4.6%. At Visit 1, the mean level of cortisol was 0.22 ng/50 mg hair (SD 0.19), and at Visit 3, this was 0.18 ng/50 mg hair (SD 0.09). A paired *t*-test showed there was no statistical difference between these values (df 22, t = 0.695, *p* = 0.5); see Figure 2.

## 4. Discussion

This project used a one-group pre-test–post-test design to assess the effects of a community-based trampoline exercise intervention on overall QoL after a burn injury in children. In our study, children demonstrated a significant improvement in bounce time between Visit 1 and Visit 2 (*p* < 0.0001), indicating better performance and skill development in trampolining across the intervention, but there were no statistically significant improvements in strength or fitness as indicated by MET scores and heart-rate reserve and recovery. This was likely due to the absence of resistance training exercises, the medium intensity of aerobic stress, and the low frequency of the sessions. Grip strength was lower than healthy norms at all visits, which is consistent with our previous work [15]. It showed an improvement after the intervention was complete, suggesting that trampolining exercise itself did not influence grip, but that children might have been more physically active after the intervention. Supporting this, the physical function scores of the PedsQL were reported as excellent by children and caregivers at all visits and did not change across visits. However, the primary aims of the study were to assess the psychosocial components of a community-based exercise, to build confidence for both children and caregivers for the child’s return to exercise, and to provide a fun group activity that also provided some peer support.

The child’s strengths and difficulties were reported by parents. When compared against the norms for Australian children, mean group scores were higher than Australian norms for emotional difficulties (Visits 1 and 3), hyperactivity (Visits 1 and 2), and total difficulties (Visit 1), but were similar to norms for peer problems, conduct, and prosocial behavior. However, when each child’s individual scores were linked across visits, median scores did not change over time.

Parents reported their child’s emotional function via the PedsQL as poor prior to the intervention, reporting scores below the minimum critical cut off score of 76 at Visit 1 [17]. They then reported significant improvements in this metric between Visit 1 to Visit 2 (*p* = 0.011), with this positive perception continuing across all visits (*p* = 0.024). This finding was mirrored in the emotional domain of the SDQ, which although stable when individually matched over time (likely due to variation within individuals), was significantly lower than population norms (*p* = 0.0048) at Visit 1 but had resolved by Visit 2. However, children did not agree. They rated their emotional functions as good prior to the intervention with no significant changes across visits (*p*-values all ≥0.1). Supporting this, hair cortisol levels did not change over time. This disparity in parent scores and child scores for the psychosocial domain of the PedsQL has been reported previously [37]. The discrepancy between the child’s reported emotional state and the parents perception of it is interesting. The reasons for this are not clear from this study; are parents’ perceptions incorrect, clouded perhaps by their feelings around the burn, or is it possible that children do not report poor emotional states in an attempt to reduce the burden on their parents, despite parents being aware? Future qualitative research might explore this.

In terms of parental post-traumatic stress symptoms, there were significant reductions in avoidance, hypervigilance, and intrusion scores on the Impact of Event Scale (IES-R). Parental avoidance scores significantly decreased from Visit 1 to Visit 2 and across all visits, while hypervigilance scores also showed a significant reduction from Visit 1 to Visit 2 and across visits. Similarly, intrusion scores significantly declined from Visit 1 to Visit 2 and over time. These results indicate that the intervention improved their emotional outlook, and these changes were maintained up to three months after the conclusion of their child’s trampolining intervention. The intervention might help parents introduce their child to safe, controlled activities, challenging their trauma-driven beliefs about ongoing danger or the risk of future accidents. The relationship between higher post-traumatic stress symptoms levels for parents [10] and their belief that their child’s emotional function is poor is consistent with previous pediatric burn research [12]. Our findings are consistent with a 12-week hospital-based wellness and exercise program which reported that parents perceived the program as helping with their children’s social–physical functioning, mental health, and psychosocial functioning [23]. Additionally, a yoga intervention with pediatric burn survivors resulted a decreased cognitive and somatic anxiety in the children [24].

This suggests that exercise interventions for children after burn injuries vicariously benefits parents more than children. We know that parents’ emotions after a burn can affect their child’s recovery [38,39]. Lowering parental anxiety may have a significant impact on a child’s confidence to return to exercise, because children often look to their parents for emotional cues and reassurance, especially during recovery. When parents are less stressed and anxious, they are more likely to provide a calm, supportive environment that encourages the child to engage in physical activities without fear or hesitation. This emotional stability fosters a sense of security and confidence in the child, empowering them to take on physical challenges and re-engage in activities like exercise with greater ease [40].

The outcomes of this study demonstrate trampolining is an easily accessible, enjoyable, safe, and appropriate therapeutic alternative for the physical and psychosocial rehabilitation of children. It is not straightforward to ascertain why the caregiver’s post-traumatic stress symptoms improved; this could have been due to them perceiving improvements in their child’s psychosocial function, or it could have been due to the peer support that caregivers might have experienced when watching their child’s sessions from the viewing gallery with other caregivers of children who had sustained a burn. The diverse cultural backgrounds of participant families might influence parental responses not accounted for here.

Another important domain demanding attention when designing a rehabilitation program for pediatric burn survivors is the opportunity to encourage healthy social interactions. When developing programs for burn survivors, acknowledging the significance of their relationships, promoting peer interactions, and boosting their self-esteem is important [40,41]. Social interactions and activities are vital to promote social recovery [25]. In a qualitative study, children voiced their worries about appearance of the scars, having to explain their scars during social interactions and worrying over how others will respond to them [9]. Therefore, engaging in programs where they can connect and relate with the other participants and not feel as if they are under-performing compared to others could be beneficial in boosting self-esteem and motivation [24], essentially fulfilling the basic human psychological needs: autonomy, relatedness, and competence, according to the self-determination theory [42].

Engaging in physically active leisure programs has proven to be beneficial for children in multiple ways. Leisure activities allow down time for children to release their emotions, enjoy, relax and develop a sense of competence [24]. Moreover, leisure involving physical activity and structured extracurricular activities improve children’s physical health, mental health, QoL, happiness, social and academic self-conceptualization, self-worth, academic performances, and importantly, positive peer experiences [43]. Further, it is important to offer the “right amount” of challenge to children, and to provide a structured, customized program in which children can readily engage [24].

In a systematic appraisal of the available literature on exercise programs for adult and pediatric burn survivors, it was highlighted that the majority of the pediatric exercise programs were in-hospital standard-of-care treatments by occupational therapists and physiotherapists, and the majority of participants had moderate or severe burn injuries. Evidence of the review indicated the importance of implementing community-based programs which included smaller burn injuries as well which will allow a comprehensive comparison with widely available hospital-based programs [20]. A benefit of our study was that the program was facilitated in a community setting, rather than the hospital environment. Community-based interventions create a more inclusive, collaborative environment for children to participate in rehabilitation programs in a real-world setting rather than in a regular hospital setting [24,44]. This change in environment is important for pediatric burn survivors as they may get traumatized and exhausted with long acute admissions and ongoing visits for scar management in the hospital. Moreover, community-based health-promoting programs in other patient cohorts, such as cerebral palsy, at risk of weight-related health problems, and youth with physical disabilities, delivered promising results in terms of increased physical activity and leisure participation [24].

Trampolining is a popular, enjoyable activity among children and can have transformative effects on human health [45]. The major physical benefits identified are a 7.82% increase in VO2max compared to 2.34% in running, losing weight, decreasing blood glucose levels, increasing bone strength and improving balance. Additionally, improvements in mental health, including reduced daytime sleepiness, controlled anxiety, reduced stress, and improved QoL were also reported. Safe trampolining interventions under suitable supervision have been studied in other populations, and the benefits are well established in the literature. Rebounding on mini-trampolines has been proven to benefit the motor skills and balance skills of children, improve bone density, and enhance mental health conditions such as anxiety, depression, and stress. Trampolining has been reported to improve physical function, cognition, and mood for patients with attention deficit hyperactivity disorder, those on the Autism Spectrum, and children with intellectual disabilities, developmental conditions, and Down Syndrome [45,46]. An adaptive bungee trampolining intervention for children with cerebral palsy resulted in improved outcomes in their lower-limb muscle strength, balance, and functional ability. Participants and their parents reported high self-rated enjoyment and satisfaction [47]. The psychosocial benefits found with this type of exercise plan are important after burn injury, and alone justify the need for this type of intervention.

This study has some limitations to be acknowledged. Firstly, recruitment was slow because parents needed to be able to attend the trampoline gym with their child, with location of residence and other commitments hindering that ability. The small sample size of 24 participants limited the statistical power of the quantitative analysis. As a result, the study was underpowered for detecting smaller effect sizes, which may have led to non-significant results even if real differences existed. Due to this limitation, we were unable to conduct robust parametric analyses, and instead, we relied on non-parametric methods such as medians and interquartile ranges. While appropriate for small samples, these methods may not fully capture the complexity of the data. Furthermore, the limited sample size precluded the use of more sophisticated statistical techniques, such as regression analysis, which would have allowed for the adjustment of potential confounding variables. The one-group, pre-post test design and small sample size indicates that we were unable to control for other factors that might influence the outcomes, including potential confounders, and this reduces the generalizability of our findings. These participants were those keen to take part in a trampolining intervention, and thus might not have included patients with greater need.

Future research that includes an extended follow-up period to assess longer-term benefits, such as improved grip strength and BMI percentiles, is needed. The lack of physical improvement in this study could be addressed with a more intensive program in terms of frequency, duration, and effort; however, it is important to note that psychosocial benefits can be detected when physical benefits cannot. Future studies with larger sample sizes will be important to confirm our results and allow for a more detailed exploration of the relationships between variables, including the possibility of adjusting for covariates in multivariable models. The covariates could include burn severity (tbsa, depth), burn location on the body (critical areas such as hands, feet, face, and genitalia). Randomized controlled studies could provide a non-exercise +/− other-exercise comparison group(s), with the advantage that propensity analysis could be performed to balance the data in terms of confounding covariates. Additionally, exploring parental feelings to their child’s exercise participation post burn could be researched using both quantitative and qualitative methods. Future research could also include nutritional aspects of recovery, given the relationship between post-burn metabolic changes, strength, and recovery.

## 5. Conclusions

The study found that, while there were no significant changes in physical measures for the children, parents reported improved emotional function in their children and experienced notable reductions in their own post-traumatic stress symptoms levels, including decreased avoidance, hypervigilance, and intrusion. This improved parental coping may have positive effects on children’s confidence in returning to exercise, even if the children’s self-reports did not reflect these emotional gains.

## Figures and Tables

**Table 1:** Longitudinal outcome analysis.

Variable	Visit 1 Median (IQR) Range	Visit 2 Median (IQR) Range	Visit 3 Median (IQR) Range	Rank Sum Test: Pre-Test to Post-Test (*p*-Value)	Rank Sum Test: Post-Test to 3 m Follow-Up (*p*-Value)	Skillings–Mack Test: Across All Visits (*p*-Value)
**MET score**	5479 (2902) 15,901	5263 (1413) 9641	6292 (3034) 16,920	0.8	0.2	0.6
**Grip Strength**	13.2 (10.5) 30.7	12.8 (7.5) 31.7	13 (8.3) 26.7	0.6	0.025 *	0.1
**10-Bounce Time**	7.22 (1.75) 5.05	8.57 (2) 5.28	Not applicable	<0.0001 *	not applicable	not applicable
**BMI Percentile**	78 (51.2) 87.6	79.5 (57) 94.2	71 (57.6) 94.2	0.1	0.7	0.021 *
**HR Reserve**	61 (27) 82	64.5 (22.5) 92	65 (42) 153	0.6	0.2	0.2
**HR recovery at 1 min**	26 (31.5) 75	33.5 (24) 79	22 (31) 135	0.5	0.1	0.4
**HR recovery at 2 min**	58 (33) 109	49.5 (23.5) 97	37 (51) 156	0.6	0.3	0.7
**HR recovery at 3 min**	57.5 (37.5) 105	48 (31) 96	43 (53) 143	0.8	0.4	0.5
**Parent-reported Physical Function Score (PedsQL)**	89.1 (18.8) 65.6	82.8 (25) 65.6	90.6 (25) 59.4	1	0.6	1
**Parent-reported Psychosocial Function Score (PedsQL)**	74.2 (29.2) 55	74.2 (30) 58.3	76.7 (21.7) 56.7	0.4	0.8	0.5
**Parent-reported Emotional Function Score (PedsQL)**	62.5 (42.5) 75	75 (30) 65	80 (30) 65	0.011 *	0.4	0.024 *
**Child-reported Physical Function Score (PedsQL)**	90.6 (12.5) 31.3	90.6 (18.8) 28.1	90.6 (15.6) 37.5	0.4	0.7	0.8
**Child-reported Psychosocial Function Score (PedsQL)**	80.8 (21.7) 53.3	80 (15) 43.3	83.3 (18.3)	0.2	0.2	0.1
**Child-reported Emotional Function Score (PedsQL)**	72.5 (45) 75	82.5 (35) 65	80 (35) 60	0.3	0.3	0.3
**SDQ Total Difficulty Score**	12.5 (6.5) 27	10.5 (8) 22	9 (11) 25	0.1	0.7	0.4
**SDQ Emotional Difficulty Score**	3 (5) 9	2 (3) 8	3 (5) 8	0.1	0.1	0.3
**SDQ Conduct Score**	2 (2) 5	2 (1) 4	2 (2) 5	0.7	0.7	0.6
**SDQ Hyperactivity Score**	4 (3.5) 10	4 (2) 10	4 (2) 10	0.1	0.8	0.5
**SDQ Peer Score**	2 (2.5) 6	1 (3) 7	2 (3) 6	0.1	0.7	0.4
**SDQ Prosocial Score**	9 (4) 6	8.5 (3) 6	8 (3) 6	1	0.5	0.8
**SDQ Total Internalizing Score**	5.5 (6) 14	3.5 (5.5) 13	4 (7) 12	0.1	0.2	0.4
**SDQ Externalizing Score**	6 (4) 15	6 (3) 13	5 (4) 15	0.3	0.6	0.4
**CPSS Symptom Score**	5 (8) 19	2 (7) 21	2 (8) 9	0.7	0.1	0.7
**IES-R Avoidance Score (parent)**	0.81 (1.13) 2.3	0.37(0.88) 3	0.13 (0.75) 1.87	0.024 *	0.6	0.009 **
**IES-R Hypervigilance Score (parent)**	0.5 (0.92) 1.5	0.17 (0.5) 2.67	0.33 (0.67) 1.5	0.005 **	0.3	0.007 **
**IES-R Intrusion Score (parent)**	0.75 (1.11) 1.5	0.5 (0.5) 3.13	0.38 (0.75) 1.5	0.020 *	0.3	0.026 *
**IES-R Total Score (parent)**	2.19 (2.5) 5.13	1.08 (1.67) 8.79	1.04 (2.08) 4.5	0.003 **	0.6	0.050 *

* *p* < 0.05, ** *p* < 0.01.

**Table 2:** Mean comparison of parent-rated Strengths and Difficulties Questionnaire scores to norms.

		Mean (SD)	Norm Mean (SD)	*p*-Value
**Visit 1**	SDQ Total Difficulty Score	11.96 (6.56)	8.2 (6.1)	0.0025 **
	SDQ Emotional Difficulty Score	3.25 (2.92	2.1 (2.0)	0.0048 **
	SDQ Conduct Score	1.79 (1.50)	1.5 (1.6)	0.4
	SDQ Hyperactivity Score	4.67 (2.58)	3.1 (2.4)	0.0014 **
	SDQ Peer Score	2.25 (1.80)	1.9 (1.9)	0.4
	SDQ Prosocial Score	8.08 (2.10)	8.3 (1.7)	0.5
**Visit 2**	SDQ Total Difficulty Score	10.42 (5.59)	8.2 (6.1)	0.08
	SDQ Emotional Difficulty Score	2.42 (2.28)	2.1 (2.0)	0.4
	SDQ Conduct Score	1.75 (1.11)	1.5 (1.6)	0.4
	SDQ Hyperactivity Score	4.29 (2.58)	3.1 (2.4)	0.0150 *
	SDQ Peer Score	1.96 (2.12)	1.9 (1.9)	0.9
	SDQ Prosocial Score	8.21 (1.79)	8.3 (1.7)	0.8
**Visit 3**	SDQ Total Difficulty Score	10.52 (6.51)	8.2 (6.1)	0.07
	SDQ Emotional Difficulty Score	3.09 (2.59)	2.1 (2.0)	0.0180 *
	SDQ Conduct Score	1.61 (1.37)	1.5 (1.6)	0.7
	SDQ Hyperactivity Score	4.00 (2.37)	3.1 (2.4)	0.07
	SDQ Peer Score	1.83 (1.80)	1.9 (1.9)	0.9
	SDQ Prosocial Score	8.04 (1.92)	8.3 (1.7)	0.5

* *p* < 0.05, ** *p* < 0.01.

## Data Availability

The data are not publicly available due to HREC privacy requirements. ThiThe de-identified data presented in this study are available on request from the corresponding author. Applicants will require appropriate ethics permissions.
